# Design of field real-time target spraying system based on improved YOLOv5

**DOI:** 10.3389/fpls.2022.1072631

**Published:** 2022-12-19

**Authors:** He Li, Changle Guo, Zishang Yang, Jiajun Chai, Yunhui Shi, Jiawei Liu, Kaifei Zhang, Daoqi Liu, Yufei Xu

**Affiliations:** ^1^ College of Mechanical and Electrical Engineering, Henan Agriculture University, Zhengzhou, China; ^2^ Changyuan Branch, Henan Academy of Agricultural Sciences, Xinxiang, China

**Keywords:** target spraying, precision weeding, machine vision, YOLOv5, lightweight model

## Abstract

Deep learning techniques have made great progress in the field of target detection in recent years, making it possible to accurately identify plants in complex environments in agricultural fields. This project combines deep learning algorithms with spraying technology to design a machine vision precision real-time targeting spraying system for field scenarios. Firstly, the overall structure scheme of the system consisting of image acquisition and recognition module, electronically controlled spray module and pressure-stabilized pesticide supply module was proposed. After that, based on the target detection model YOLOv5s, the model is lightened and improved by replacing the backbone network and adding an attention mechanism. Based on this, a grille decision control algorithm for solenoid valve group on-off was designed, while common malignant weeds were selected as objects to produce data sets and complete model training. Finally, the deployment of the hardware system and detection model on the electric spray bar sprayer was completed, and field trials were conducted at different speeds. The experimental results show that the improved algorithm reduces the model size to 53.57% of the original model with less impact on mAP accuracy, improves FPS by 18.16%. The accuracy of on-target spraying at 2km/h, 3km/h and 4km/h speeds were 90.80%, 86.20% and 79.61%, respectively, and the spraying hit rate decreased as the operating speed increased. Among the hit rate components, the effective recognition rate was significantly affected by speed, while the relative recognition hit rate was less affected.

## 1 Introduction

Spraying chemical pesticides is the main method of crop pest and weed control at present (Song et al., 2015; Zhang et al., 2015). Pesticides have contributed greatly to increasing agricultural production and are an essential material basis for ensuring food security (Cooper and Dobson, 2007; Popp et al., 2013; Verger and Boobis, 2013). At the same time, the excessive use of pesticides has also brought many problems, such as pesticide waste, chemical residues, environmental pollution, etc. The efficient and scientific use of pesticides is an inevitable requirement to achieve high-quality agricultural development (Gil and Sinfort, 2005; Pan et al., 2019). The current main method of pesticide application is continuous and continuous spraying of pesticides with full coverage of the field. Most pesticides enter the natural environment through evaporation, surface runoff, infiltration into the soil, etc., and only a small portion falls on the spraying target to be used effectively (Zabkiewicz, 2007; Zheng et al., 2018). Especially when weeding in the seedling stage of crops, the weed canopy is small, the bare land occupies the main area, and most of the pesticides fall into the soil. In China, for example, according to relevant data, The effective utilization rate of pesticides for major food crops in China is only 40.6% in 2020 (Lan et al., 2021). Target spraying is an effective technique to improve the utilization rate of pesticides. Sensors sense the position and size of the target in the field and control the opening and closing of the nozzle to accurately spray the target, which can significantly improve the effective utilization rate of pesticides and reduce the use of pesticides (Yu et al., 2019).

Detection of targets in the field is the key to target spraying technology (Song et al., 2015). There are machine vision solutions and non-machine vision solutions for target detection of target application systems based on real-time sensor technology. (Meshram et al., 2022). Ultrasonic and laser sensors can be used to detect the position of plants, using the time it takes for the ultrasound or laser to reach the target and reflect back, and then calculating the distance to the target. Over the years, related researchers have carried out a series of explorations in this field, Chueca et al. (2008); Palleja and Landers (2015) and Zhou et al. (2021) used ultrasonic sensors to measure the size and density of trees and control orchard spray system. Deng et al. (2008) applied infrared photoelectric detection technology to agriculture to achieve crop detection and target spraying with an adjustable detection range of 0.1 to 0.5m. Geng et al. (2012) designed a tobacco-targeted application system, which uses the detection results of the photoelectric switch to control the opening and closing of the spray solenoid valve. Non-visual sensors are difficult to pinpoint the location of the target, and detection targets are dominated by tall plants such as fruit trees, rather than smaller-scale crop seedlings and weeds in fields.

In machine vision-based on-target spraying solutions, traditional image algorithms analyze by plant morphology, texture and spectral features. Dammer (2016) used the different wavelengths reflected by the plants and the land in the field, distinguishes the vegetation and the land through cameras and filter lenses, and realizes the real-time variable spraying of herbicides in the carrot field. Esau et al. (2018) designed a machine vision pesticide variable spraying system that distinguishes weeds by the difference in color between green weeds and reddish-brown wild blueberries when herbicides are applied in spring and fall. Özlüoymak et al. (2019) discriminated green objects in images according to their greenness and controlled the opening and closing of sprinklers according to the coordinates of artificial weeds. Zhao et al. (2022) used the improved support vector machine classification algorithm to detect cabbage and weeds, and designed a target spray system to achieve an average effective spray rate of 92.9%. Although the traditional image processing method has been verified in the target spray system, it is primarily aimed at scenarios with relatively ideal operating environments and large target volumes, such as greenhouses and vegetable with large plant distances. For outdoor field production scenarios with complex working conditions, the targets are relatively dense and small, making it difficult to be applied in practice. At the same time, occlusion and changing lighting conditions in natural environments remain significant challenges for spray target identification and localization (Wang et al., 2019).

In recent years, deep learning-based target detection techniques have led to substantial improvements in the accuracy of target recognition. Classic target detection algorithms include R-CNN, Faster-RCNN, SSD, Mask RCNN, YOLO series algorithms, etc. (Girshick et al., 2014; Liu et al., 2016; Redmon et al., 2016; He et al., 2017; Ren et al., 2017). In particular, the YOLO series of target detection algorithms proposed by transforming the target detection problem into a regression problem, can directly output the category and coordinate information of the predicted frame end-to-end (Redmon et al., 2016; Redmon and Farhadi, 2017; Redmon and Farhadi, 2018). Related researchers applied different object detection algorithms to weed identification in the agricultural field (Xu et al., 2021). Potena et al. (2016) used two different CNNs to process RGB and NIR images to quickly and accurately identify crops and weeds. A lightweight CNN is used for fast and robust vegetation segmentation, and then a deeper CNN is used to classify the extracted pixels between crops and weeds. Patidar et al. (2020) proposed an improved Mask RCNN model to extract early cranesbill seedlings. You et al. (2020) proposed a semantic segmentation method for weed crop detection based on deep neural networks (DNNs). Hailong et al. (2022) used the YOLOv5 algorithm to process aerial images of drones, generate prescription maps, and transmit them to sprayers to achieve fixed-point weeding. Although the YOLO algorithm has shown excellent performance in object detection, its requirements for high-performance GPUs and other devices constrain its deployment in the field (Qin et al., 2021), so it remains important to investigate further lightweight improvements to its model.

In this paper, deep learning based object detection technology is applied in the field of precise spraying, and a precise real-time target spraying system based on machine vision is studied for the field scene. Firstly, the overall design scheme of the system is proposed, including image acquisition and detection module, an electronically controlled spray module, and a pressure-stabilized pesticide supply module. Then, lightweight improvements are made based on the YOLOv5s model. On this basis, a grid decision-making algorithm is designed to determine the solenoid valve group’s opening and closing. At the same time, a common malignant weed is selected as an object to produce data sets and complete model training. Finally, the hardware system and identification model are completed to be deployed on the electric boom sprayer, and field experiments are conducted at different speeds. The experimental results show the accuracy of the application of the designed system, which can effectively improve the effective utilization rate of pesticides, reduce the amount of pesticide used, and mitigate the environmental pollution of farmland, which has certain significance for agricultural production intelligence and ecological protection.

## 2 Materials and methods

### 2.1 Overall scheme design of real-time target spraying system

**Figure 1:**
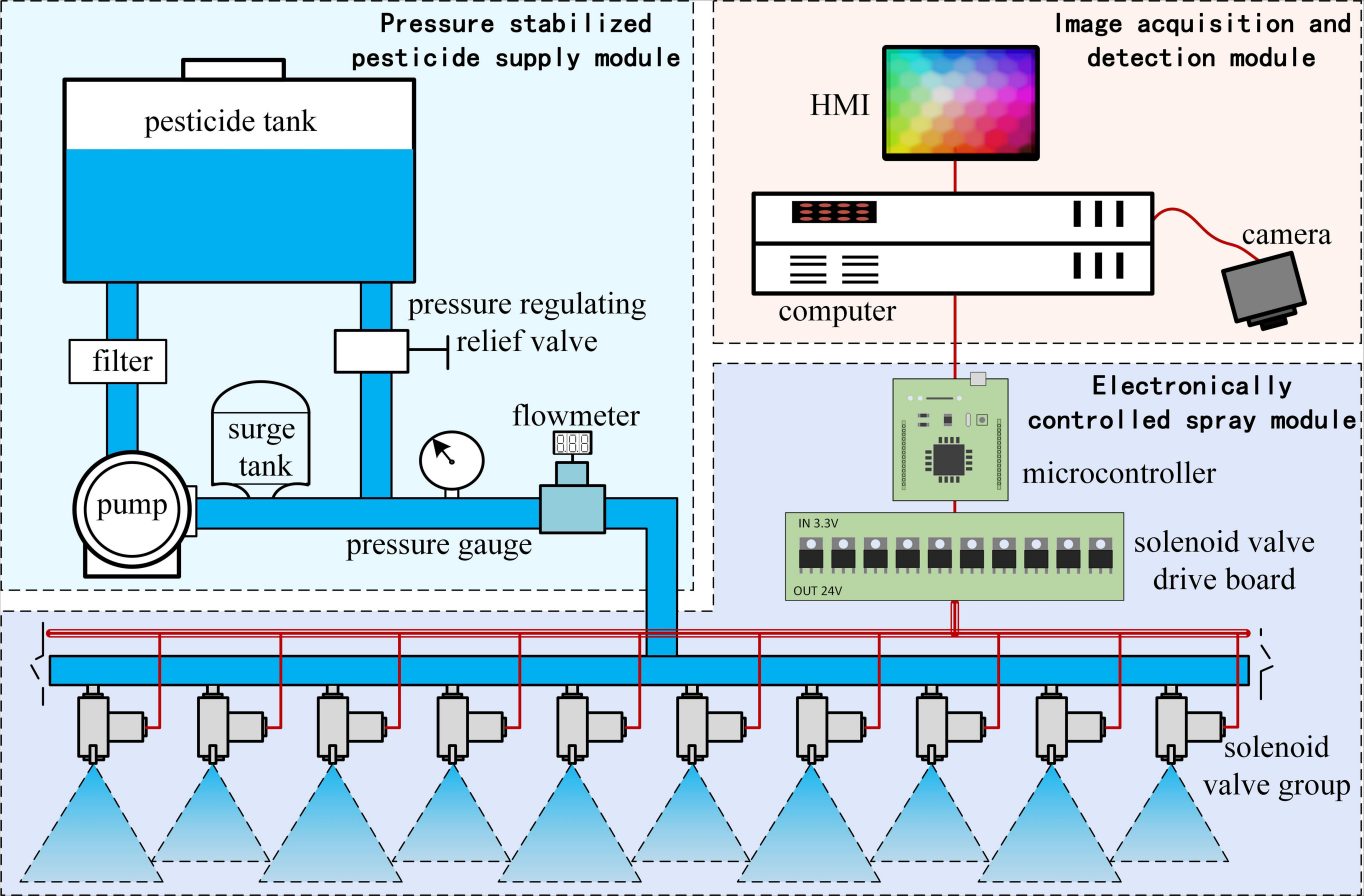
The components of the real-time target spaying system.

The main work of this research is to develop a set of real-time target spraying system based on deep learning, which can identify crops or weeds in real time and spray chemical agents on the spraying target, thereby greatly reducing the use of pesticides. The real-time target spray system is mainly composed of an image acquisition and detection module, an electronically controlled spray module, and a pressure-stabilized pesticide supply module. The system structure is shown in **Figure 1**.

The image acquisition and recognition module comprise an industrial camera and an onboard computer. The industrial camera is responsible for collecting image data. The onboard computer, as the upper computer of the system, is responsible for tasks such as processing image data and decision-making of opening and closing of the solenoid valve group. Among them, the industrial camera is a 2-megapixel camera with a 2.8-12mm zoom lens, a 1920×1080 video image resolution, and a 30FPS frame rate. The onboard computer CPU is Intel i7-1165G7, the memory is 16GB, and the graphics card is NVIDIA RTX2060. The industrial camera transmits image data to the computer *via* a USB interface. The mounting height of the camera is determined by the size of the field of view. The lower the mounting height, the smaller the camera field of view and the larger the scale of the ground target in the image, but more camera combinations are needed to cover the entire operating width of the sprayer. Conversely, the higher the mounting height, the larger the camera field of view, but the smaller the scale of the ground target in the image. In this study, two cameras were mounted on the spray bar, each governing half of the operational width. The operating width of the experimental prototype was 3.3m, and the installation height was determined by field adjustment to be 1m above the ground, with each pixel corresponding to a size of 0.859mm on the ground.

The electronically controlled spray module consists of a microcontroller, a solenoid valve group, a solenoid valve driver board, and a nozzle set. The microcontroller belongs to the lower computer, and is responsible for receiving signals from the upper computer and controlling the action of the solenoid valve group. The microcontroller model is Nucleo F411RE, 128kB RAM, 512kB Flash, maximum main frequency is 100MHz. The solenoid valve group is composed of high-frequency solenoid valves, the model is suodi2V025, the working voltage is DC 24 V, the working pressure range is 0∼1.0MPa, and the maximum operating frequency is 10Hz. In the non-energized state, the solenoid valve is in a closed state under the action of the spring. After the solenoid coil is energized, the valve core is pulled in momentarily, and the solenoid valve is switched to the conducting state. The output voltage of the microcontroller pins is 3.3V, which could not drive the solenoid valve directly, so MOS tubes are used to build the solenoid valve driver module to convert the 3.3V signal to 24V level. In order to prevent the load from interfering with the microcontroller, a photoelectric coupling element is designed and arranged at the front of the MOS tube to convert the electric signal into an optical signal, and then convert the optical signal into an electric signal, which is used to isolate the direct connection between the microcontroller and the solenoid valve group in the circuit. The nozzles are fan-shaped nozzles with a fog surface angle of 25°, and the spacing between nozzles is 15cm.

**Figure 2:**
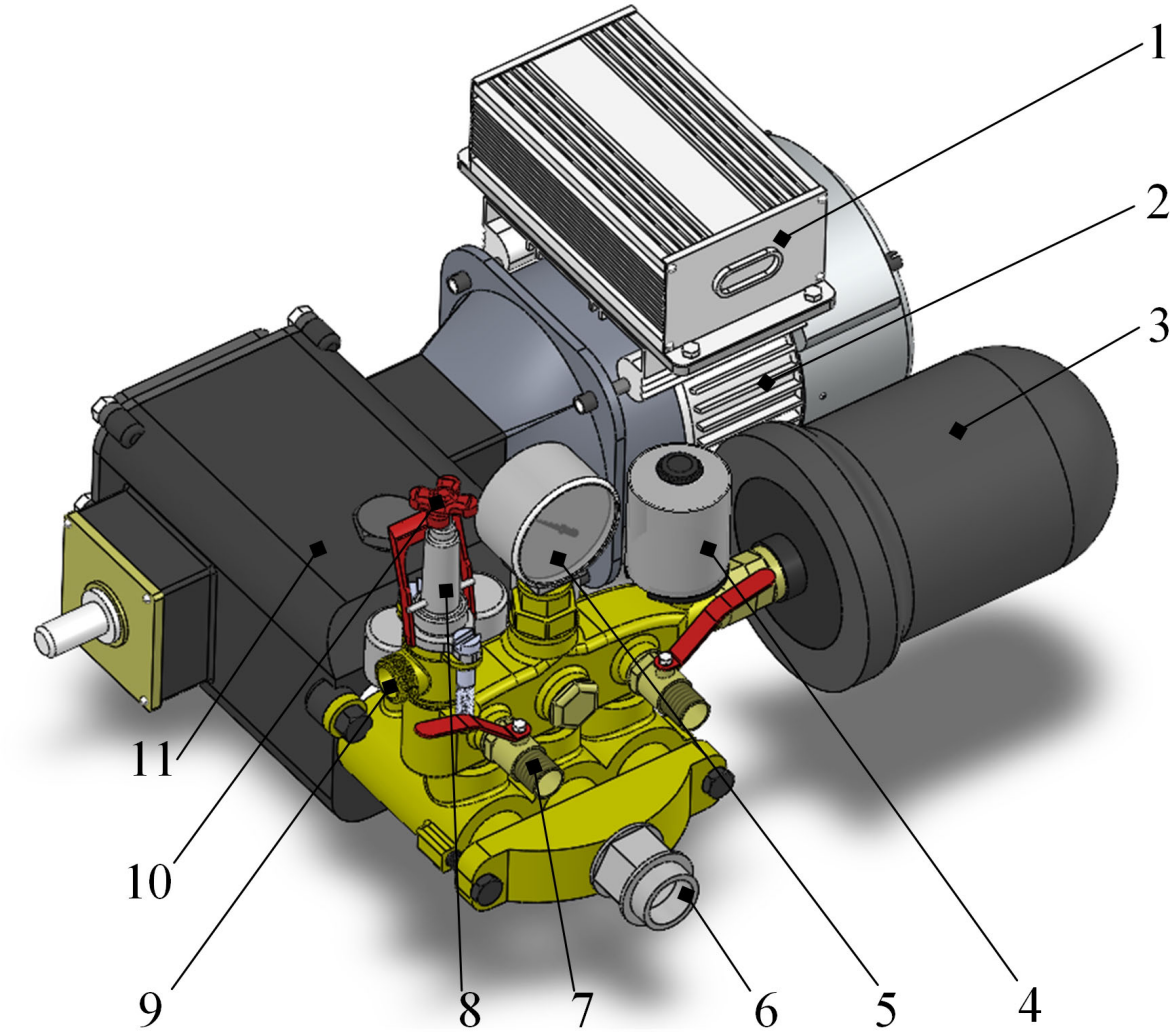
Electric pressure stabilizing pesticide pump. 1. Motor Controller 2. Drive motor 3. Surge tank 4. Pressure switch 5. Pressure gauge 6. Pesticide inlet 7.Pesticide outlet 8.Pressure regulating valve 9. Pesticide return outlet 10.Pressure regulating handwheel 11. Pump body.

The pressure-stabilized pesticide supply module includes a pesticide tank, electric plunger pump, pressure gauge, flowmeter, and pressure regulating relief valve. Since the spray on the target is intermittent, it will cause strong pressure fluctuations and water hammer effect in the pipeline, which will easily reduce the life of the pipeline components or even damage them. Therefore, the design of automatic pressure stabilization supply pump, as shown in **Figure 2**, two pressure regulating elements, a surge tank and a pressure switch, were added to the existing electric piston pump.The internal surge tank is a rubber capsule, which can maintain certain water pressure and absorb the pressure fluctuations caused by the sudden start and stop of the nozzle. The pressure switch is connected to the motor controller, which automatically turns off the pump when the water pressure is higher than the set shut-off pressure, and automatically absorbs when the pressure is lower than the set-on pressure and starts the pump to work. The interplay between the pressure stabilizer and the pressure switch enables the pump to supply on demand according to the amount of pesticide sprayed, avoiding idling and frequent starting and stopping of the pump.

**Figure 3:**
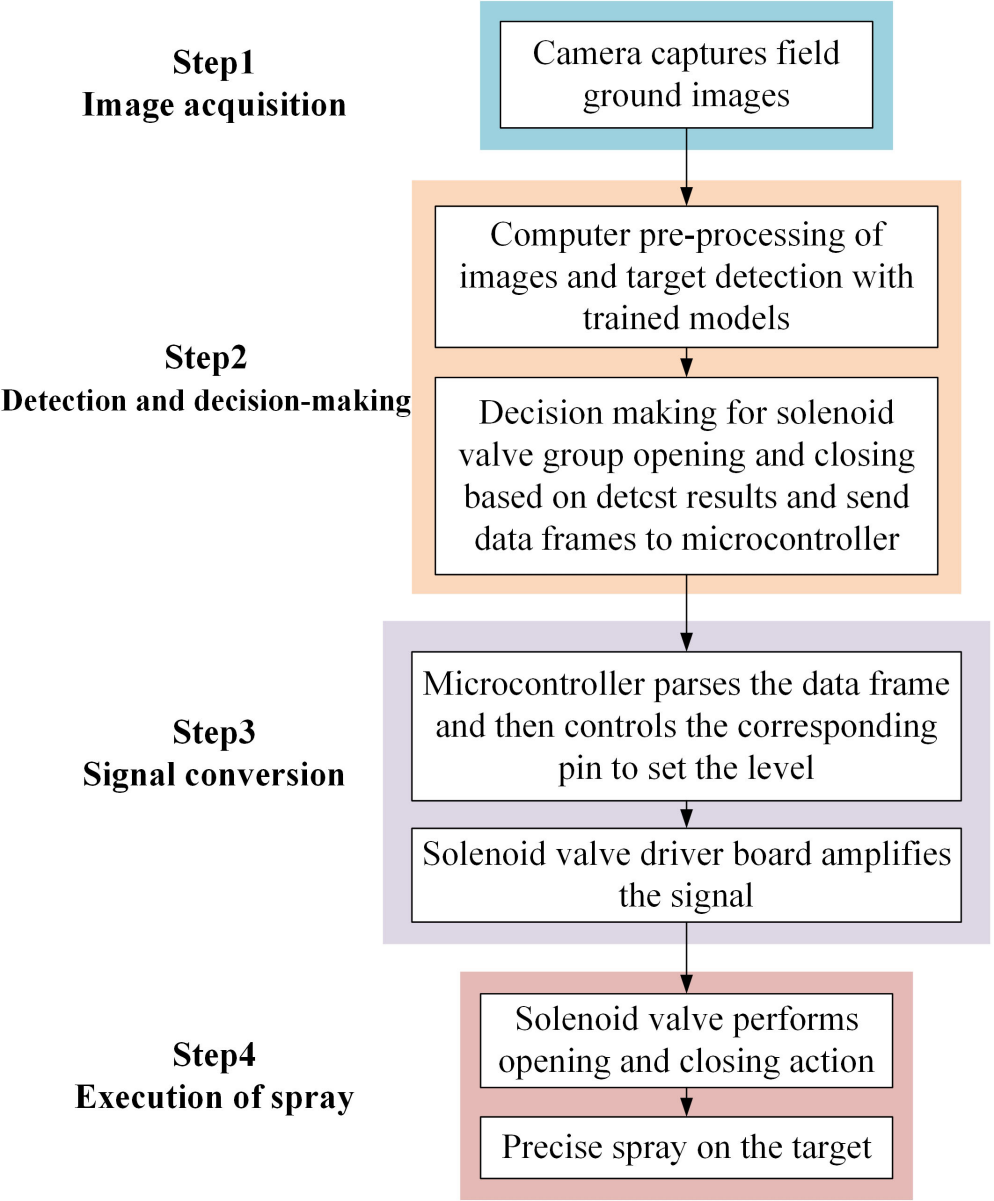
The system workflow block diagram.

The workflow of the real-time target spraying system is divided into four parts: image acquisition, identification decision-making, signal conversion, and spray execution, as shown in **Figure 3**. When the system is working, the camera collects field ground image information to the onboard computer. The computer preprocesses the image, and uses the trained deep learning model to detect the target, and then decides the opening and closing of the solenoid valve group according to the detection result. On this basis, the computer sends the decision signal in the form of a data frame to the electronically controlled spray module in real time through the serial port, and the microcontroller of the electronically controlled spray module parses the data frame and sets the corresponding control pin to the level. Finally, the solenoid valve drive board amplifies the signal and drives the solenoid valve group to open and close to complete the target spraying task. In the meantime, the surge tank and the pressure regulating relief valve of the pressure stabilized pesticide supply module work together to suppress the pressure of the pipeline caused by intermittent spraying, so that the pesticide supply pipeline always maintains a constant supply pressure, so as to ensure the quality of spray nozzle atomization.

### 2.2 Image data collection and processing

The experimental data collection site is Suixian County (*34.136°N, 115.343°E*), Henan Province, from July 16 to July 18, 2022. The collection was made from the common weed Cirsium setosum (*Cirsium arvense* var. *integrifolium*), a perennial malignant weed that is harmful to dry crops such as wheat, cotton, and soybeans, and is difficult to control due to its well-developed rhizomes and strong resistance to pesticides.

**Figure 4:**
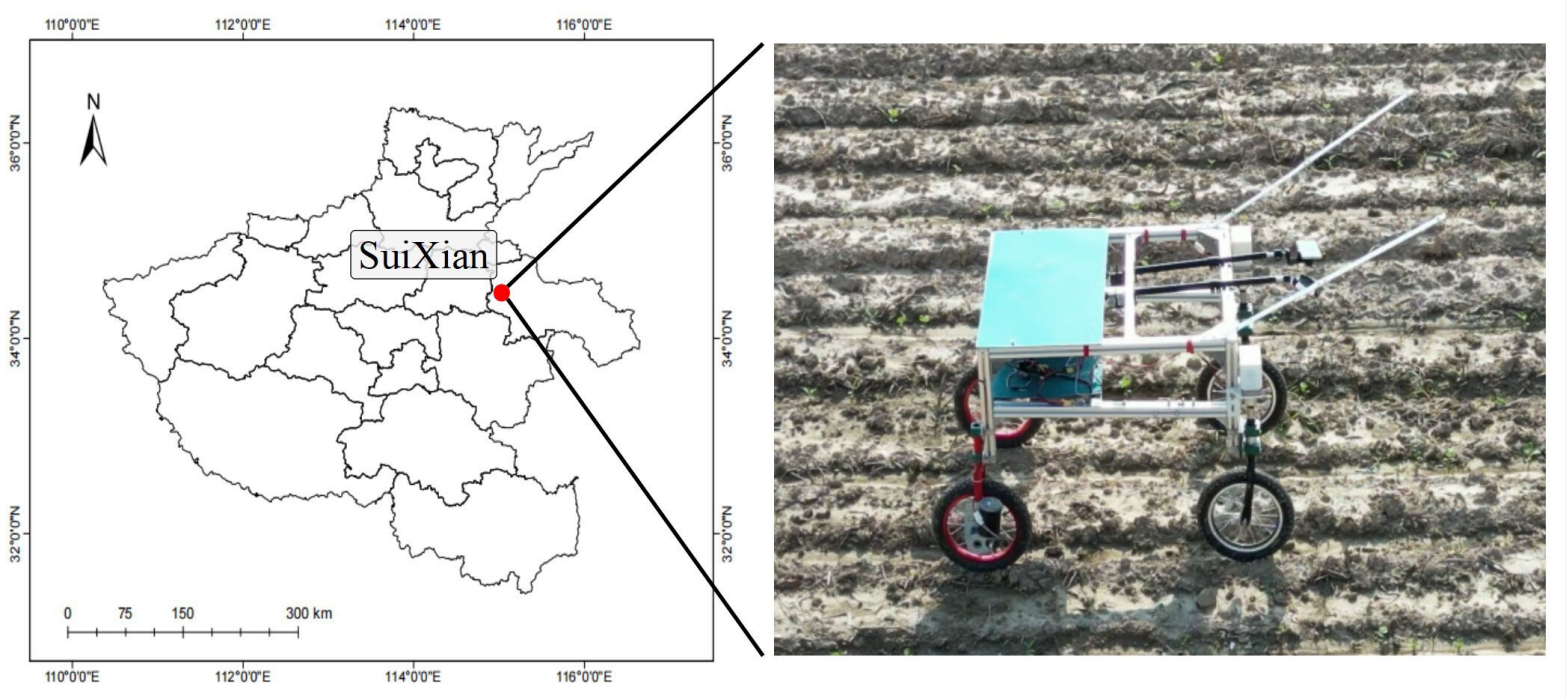
Remote-controlled field image collection vehicle.

If the collected data differs significantly from the actual application scenario, it will lead to the problem of reduced recognition accuracy of the trained model in the actual application scenario. In order to ensure the quality of the collected model training data and at the same time to improve the efficiency of data collection, a remote-controlled electric image data collection vehicle was used in the design of this study, as shown in **Figure 4**. The image collection vehicle is driven by a 12V motor, with image capture designs such as industrial cameras and mobile phones fixed to the front of the vehicle, shooting at a downward angle and at the height of 80 cm above the ground to simulate the position and state of the camera during actual operation.

A total of 3,200 pictures containing Cirsium setosum weeds were collected. After manual inspection, it can meet the needs of deep learning model training. The images were manually annotated using the image annotation tool CVAT, and the annotated data were exported in the PASCAL VOC data format. After that, the entire dataset is randomly divided into a training set, validation set, and test set according to the ratio of 8:1:1 for subsequent model training and testing.

### 2.3 Lightweight improved model based on YOLOv5

Weeds are not uniform in spatial scale size and are randomly distributed across the field, and small fluctuations up and down in recognition accuracy do not have a significant impact on pesticide spraying. Deep learning models, on the other hand, require a large number of inference calculations and demand high performance from the equipment. Therefore, the model size can be reduced by lightening and improving the network model to allow deployment on vehicle-mounted edge devices with limited computational resources and to improve the recognition frame rate to achieve local real-time target recognition for the target spraying system, while essentially not affecting the effect of on-target spraying.

#### 2.3.1 YOLO v5s model

In the field of target detection, the single-stage detection algorithm represented by the YOLO series offers a good balance between accuracy and speed (Redmon et al., 2016). YOLOv5 is one of the YOLO series networks, by transforming the target detection problem into a regression problem, the whole image is input, and the class and coordinate information of the prediction box can be directly output. It is one of the current models with the best target detection performance and has the speed of inference fast, end-to-end, and so on (Chen et al., 2022). YOLOv5 contains five scale models, N, S, M, L, and X. These five models become progressively larger, slower, and more accurate. This system selects the YOLOv5s model as the base model and optimizes and improves on it to design a lightweight field target detection model.

The YOLOv5s model consists of three parts: backbone network, neck network, and detection head. The backbone network is used to extract image features, the neck network is used to integrate the features of the entire scale, the feature pyramid is generated, and the detection head is used to regress the position and category of the output prediction box. Among them, the backbone mainly uses the CSPdarknet+SPPF structure, and Neck uses the PANet structure.The backbone network consists mainly of a CSPdarknet network of Conv modules stacked with C3 modules and an SPPF structure. Convolution, BN, and SiLU activation functions are included in Conv. The Neck part of YOLOv5s uses the PANet structure, which is a bottom-up feature extraction and fusion structure added to the FPN. After FPN upsampling for feature fusion, PANet is added, but the two feature maps are fused by splicing to obtain three feature maps of different sizes. For the YOLOv5s detection head, it performs target prediction and classification on the output three feature maps. Positive and negative samples are distinguished on the anchor box by matching across the grid cell, mainly by adjusting the offset of the predicted target centroid relative to the upper left corner of the grid, with the aim of removing sensitivity from the grid cell.The YOLOv5s model loss calculation includes classification loss, localization loss, and target loss. The total loss is calculated as follows:


(1) $$Loss=\lambda_{1}L_{cls}+\lambda_{2}L_{loc}+\lambda_{3}L_{obj}$$

where *λ*_1_, *λ*_2_, *λ*_3_ are balance coefficients.

#### 2.3.2 MobileNetv3 and attention mechanism module

The original CSPdarknet backbone network in YOLOv5 has a large amount of convolutional processing, which occupies a large amount of computing power and computation time, and is not suitable for deployment on edge computing devices with limited computing power (Wang et al., 2022). In this case, this study replaces the backbone network of YOLOv5 with the more lightweight deep learning model MobileNetv3 (Howard et al., 2019) to reduce the computational and model size of the original backbone network.

**Figure 5:**
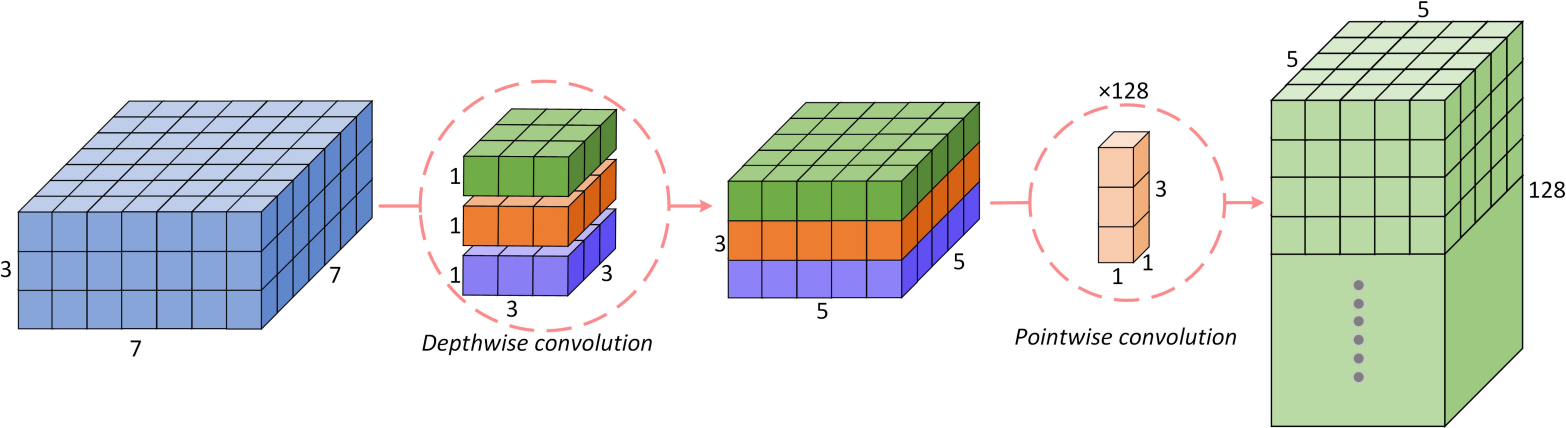
Depthwise separable convolutional structure.

MobileNet v3 is the third version of the MobileNet family of networks. The core idea of the MobileNet series of models is to replace the standard convolution operation with a depthwise separable convolution. As shown in **Figure 5**, the input information is first subjected to depthwise convolution and then pointwise convolution, which greatly reduces the amount of computation and parameters and realizes recognition. The processing speed is significantly improved with little loss of accuracy.

**Figure 6:**
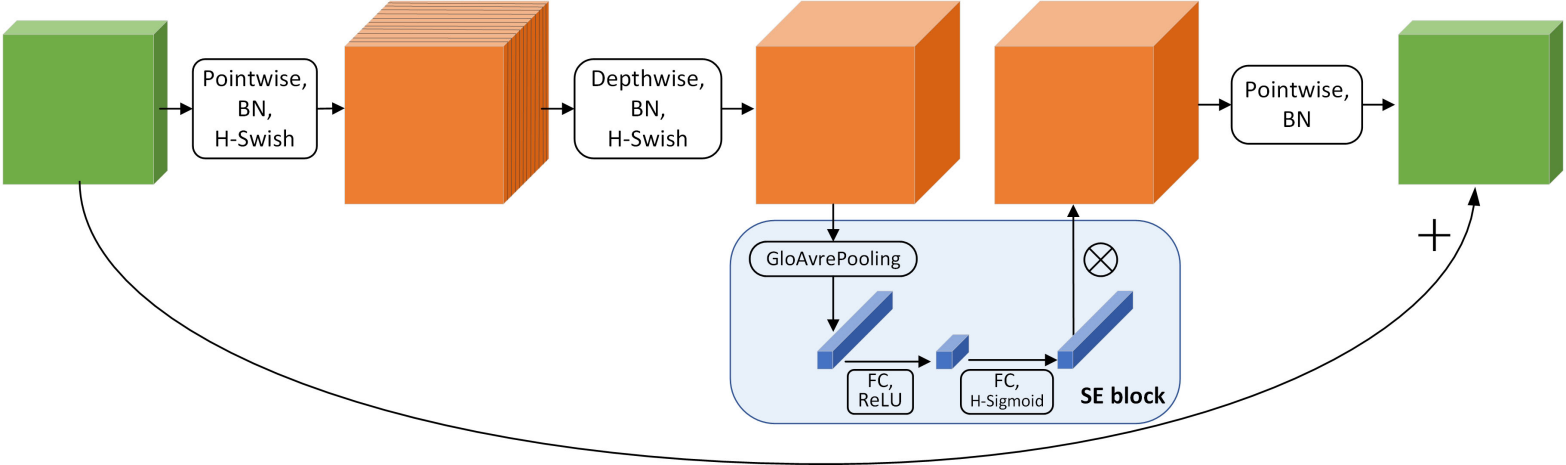
MobileNetV3 block structure.

MobileNet v3 adds a number of tricks to the depth-separable convolution to make the model more robust. First, the input matrix is increased in dimension and activated using the H-Swish activation function; The Swish activation function is activated, and an attention mechanism is added; then, pointwise convolution is used to reduce the dimension, and a shortcut connection is used between the input information and the output channel. The block structure is shown in **Figure 6**.

The attention mechanism is similar to the human visual selective attention mechanism. It selects the information that is more critical to the task goal from many pieces of information, suppresses the useless information, and increases the weight of the useful information. The core idea of the SE (Squeeze and Excitation) module is to automatically learn the feature weights according to the loss through the fully connected network so that the effective feature channel weights are increased. The learning process adaptively acquires the weights for each channel and updates the original data according to the weights, devoting computational resources to the different channels in a rational way.The structure is shown in **Figure 6**. The SE attention module first compresses the two-dimensional features (h*w) of each channel into a real number by global averaging pooling, changing the feature map from (h*w*c) to (1, 1, c). A weight value is then generated for each feature channel, and the correlation between channels is constructed through two fully connected layers, outputting the same number of weights as the number of channels in the input feature map. Finally, the normalized weights obtained earlier are multiplied channel by channel with the input feature map.

#### 2.3.3 Improved model

**Figure 7:**
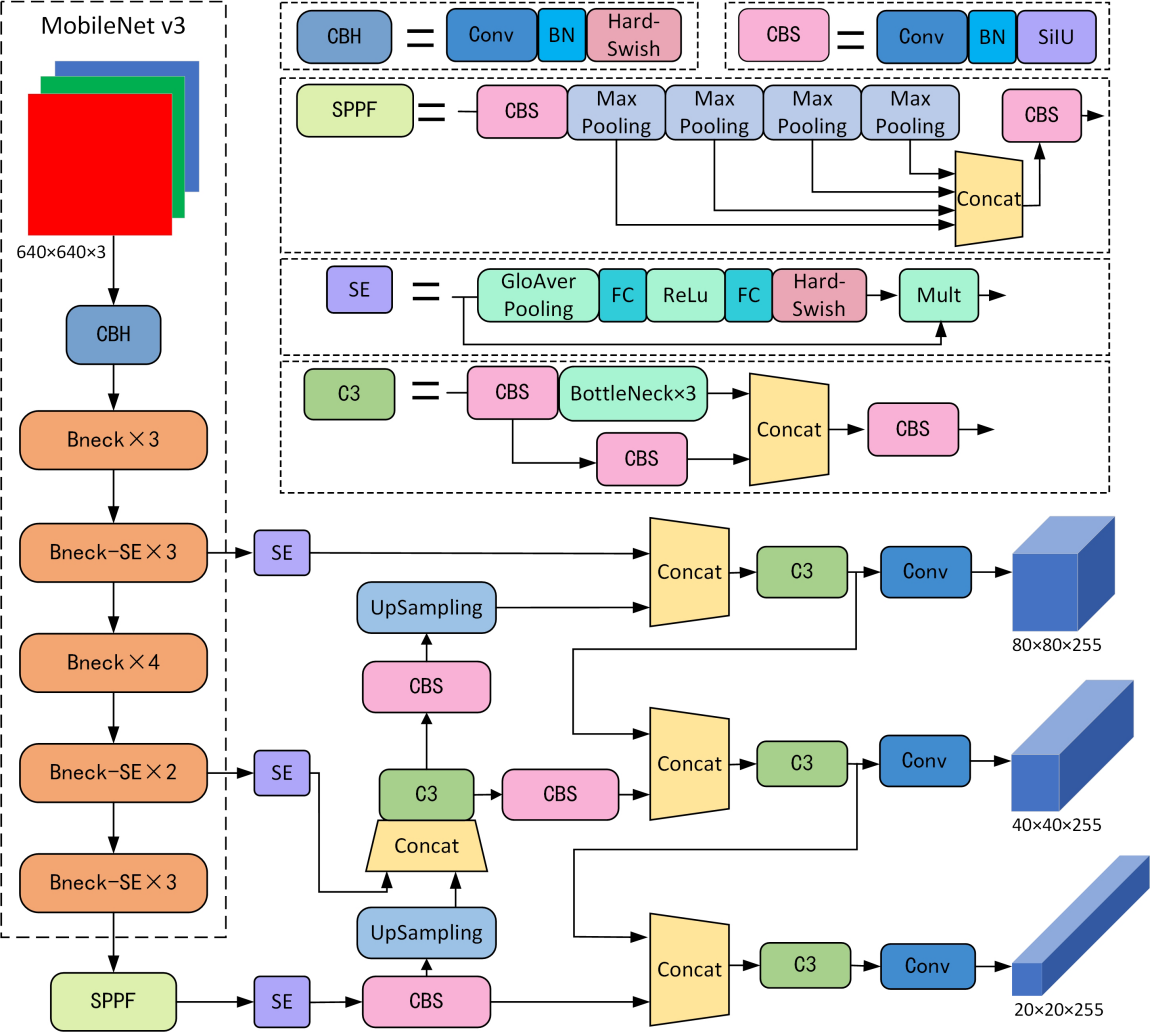
YOLOv5-MobileNetv3-SE model network structure.

In order to better balance the accuracy and speed of the model, the model can be deployed on edge computing devices with limited computing power. In this study, the backbone network of YOLOv5s is first replaced with a more lightweight MobileNet v3 network model to reduce the computation and model size of the original backbone network. Afterward, the field images are relatively complex, and some weeds have small targets, which may easily cause false and missed detection problems. In this study, SE attention modules are added behind the three outputs of the backbone network respectively, so that the network can improve the computational efficiency through automatic learning and increase the effective feature channel weights, so that the network can focus on important feature channels, thereby improving the accuracy of the model for small target weed recognition. The improved YOLOv5s model is shown in **Figure 7**.

### 2.4 A grille decision algorithm for solenoid valve group on-off

**Figure 8:**
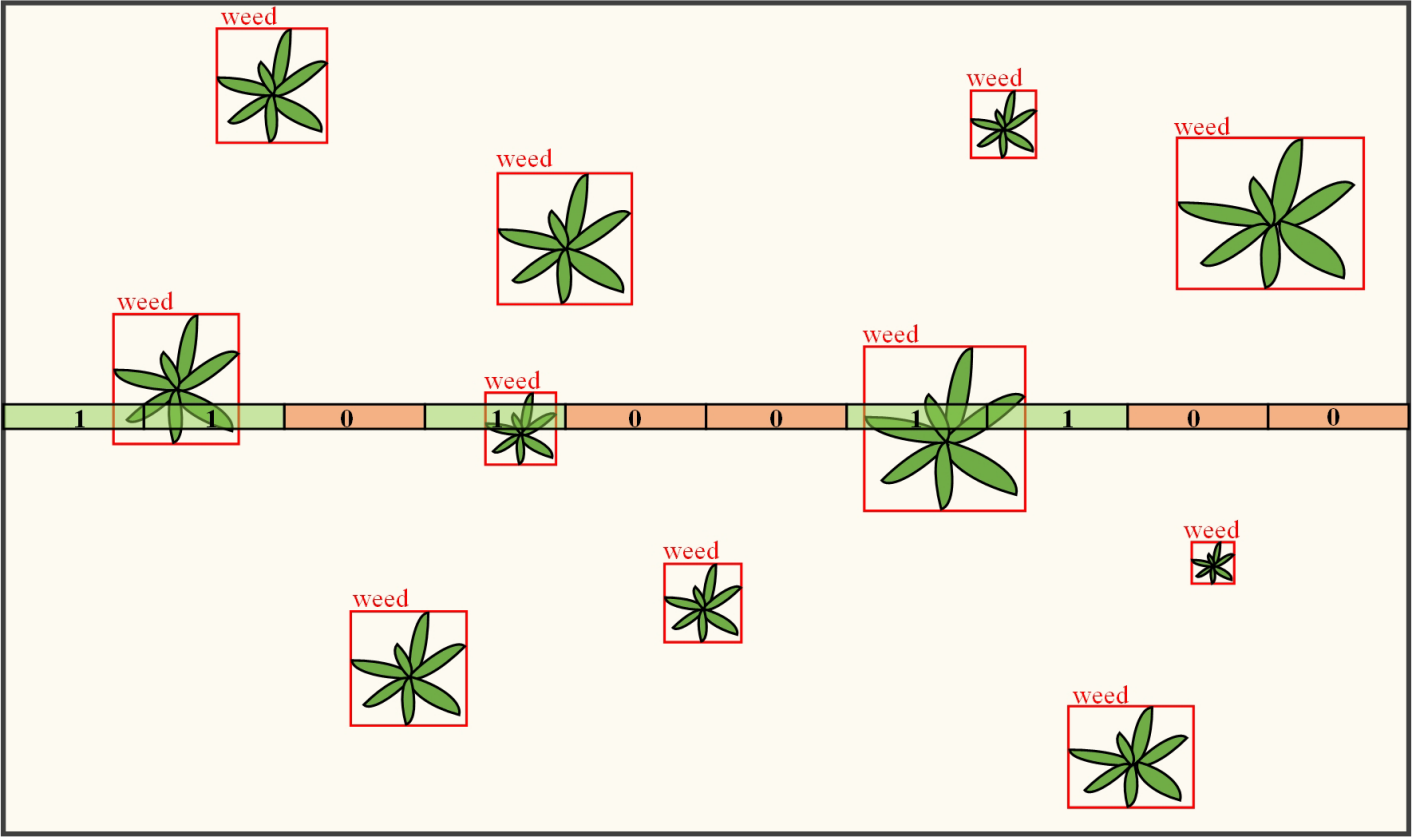
Schematic diagram of grille decision algorithm.

The weed location information identified in the image for target spraying system needs to be converted into nozzle opening and closing control information. Therefore, this study proposes a grid decision-making algorithm. The specific method is: to draw a single-layer grid on the screen, the number of grids corresponds to the number of solenoid valves on the boom, and the length of the line segment is the average spray width of each solenoid valve. As shown in **Figure 8**. As the spray bar sprayer works forward, the ground image moves down the screen, and the onboard computer also performs target detection and draws a prediction box on the target in real time. If a prediction box overlaps a grid, the target is in the spray area of the corresponding nozzle. The system detects the area of each grid intersecting the prediction box and if the area is greater than a set threshold, a decision position of 1 is made, indicating that the solenoid valve needs to be opened for spraying; otherwise, 0 is set, indicating that the solenoid valve is closed. Each grid in the grille is like a virtual touch switch that is pressed when the prediction box passes by and automatically pops up when it leaves.

Grid detection is performed on each frame of a video image to generate a decision frame composed of 0 and 1, which is then sent *via* the serial port to the microcontroller for parsing and driving the solenoid valve set on and off. After receiving the data frame, the microcontroller parses the data frame by bit and sets the level state of the corresponding GPIO pins, the output voltage of the controller is 3.3 V. After the signal amplification module is boosted to 24V, the solenoid valve group is driven to act, the nozzle and the pesticide supply line are conducted, and the pesticide is atomized to achieve target application.

For the delineated grid, which has a certain width, this allows the prediction frame to be in contact with the grid for a little longer than the solenoid valve passes through the ground target, corresponding to a slightly larger spray coverage area in the longitudinal direction. This redundancy is designed to compensate for nozzle installation errors, speed fluctuations, spray bar vibration and droplet drift in the forward direction of the machine, thus ensuring complete coverage of the ground target by the spot spray. In addition, for small weeds, the nozzle has a very short time to pass over them, and the solenoid valve can be switched off before the pesticide has had time to atomize. Setting a certain grid width to ensure that the solenoid valve has a base opening time, thus guaranteeing spraying effectiveness.

The width of the detection grille corresponding to the distance on the ground needs to be greater than the distance the vehicle advances in the time used to process one frame of the image, otherwise it will likely cause a missed detection. Therefore, the width of the grid should not be too small, and the width set in this study was 60 pixels, which corresponds to a detection width of 51.54 mm on the ground.

## 3 Experiments

### 3.1 Model training

For model training, the input image is first resized to 640 × 640 pixels, while the image padding method is used to maintain the aspect ratio of the original image. Then use the Pytorch framework to build the improved YOLOv5s model. Anchor boxes are generated using multiple iterations of the K-means algorithm. Mosaic data augmentation is also used in the training process, which further enriches the background of the detected object, enhances the network model’s cognition of weed characteristics, and enhances the robustness and generalization performance of the model. Use cosine annealing learning rate during training and optimize training with Adam optimizer. The entire model was trained for a total of 300 epochs.

**Figure 9:**
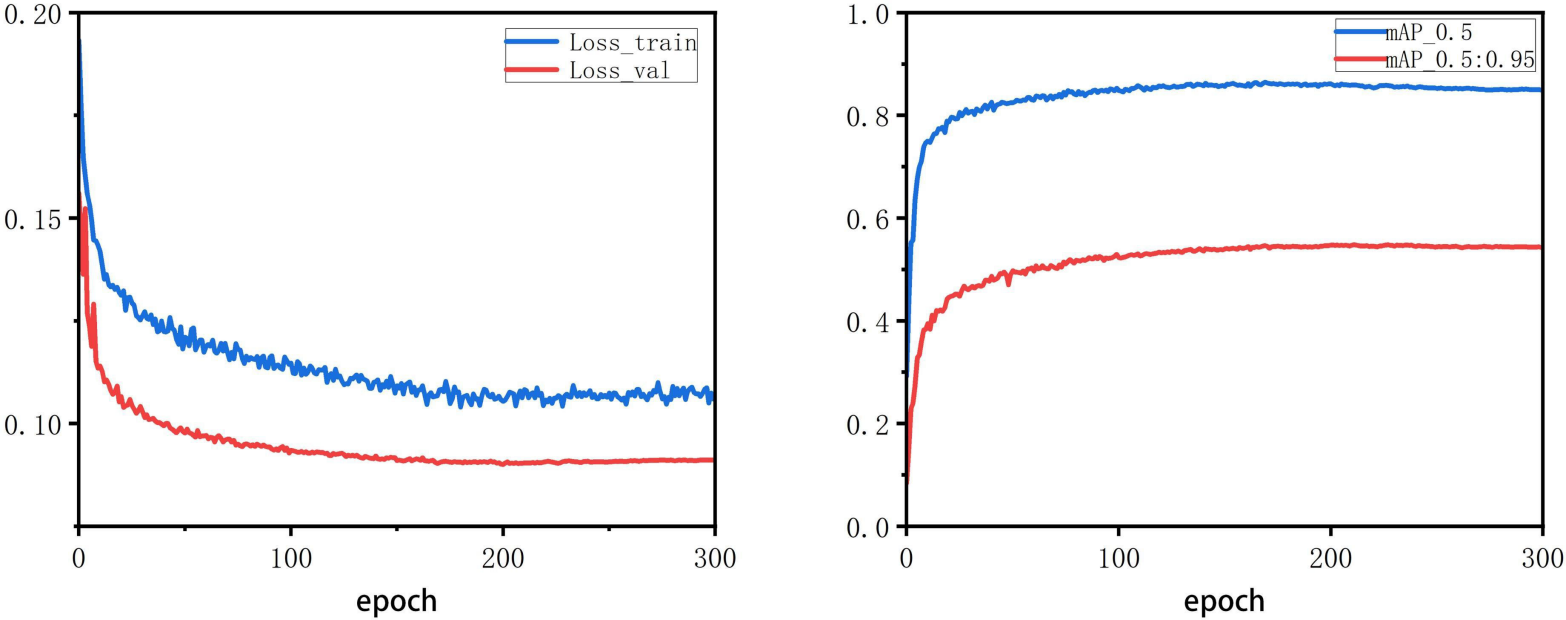
Model training convergence process.

After passing the data set into the improved YOLOv5s model, it is judged whether the model training has reached convergence by observing the change of the Loss value of the model during the training process and the change of the mAP curve on the validation set. **Figure 9** shows the training process curve. It can be seen that after 170 epochs of training, the loss of the network model gradually decreases and stabilizes, the mAP on the validation set rises and stabilizes round by round, and the training continues until the loss converges. After the model converged, the weights with the lowest loss in the last few training cycles were selected as the trained model. Finally, the model performance is tested on the test set to verify its performance respectively. To ensure the reliability and accuracy of the experiments, the tests were all completed in the onboard computer configuration environment in **Table 1**.

**Table 1:** Parameters of the experimental equipment.

Configuration	Category	Model training computer parameters	Onboard computer parameters
Software configuration	Operating systems	Windows10	Windows10
	Coding Language	Python 3.7	Python 3.7
	Code compiler	Pytorch 1.7.1	Pytorch 1.7.1
	Computing platform	CUDA 11.5	CUDA 11.5
Hardware configuration	CPU	i7-12900K	i7-1165G7
	GPU	NVIDIA RTX 3090(24GB)	NVIDIA RTX 2060(6GB)
	Memory size	64GB	16GB
	SSD	512GB	512GB

### 3.2 Model evaluation metrics

In order to accurately evaluate the identification effect of the improved model for weeds, this study adopts the precision, recall, F1 score, average precision, network parameters, model size, and detection speed indicators for evaluation. Among them, the data set of this study mainly detects typical weeds and only contains one category of weeds, so there is no need to calculate mAP results. In the evaluation experiments, the IoU threshold was set to 0.5. The formulas for precision (*P*), recall (*R*), F1 score, and average precision (AP) are as follows:


(2) $$P=\frac{TP}{TP+FP}\times 100\%$$

(3) $$R=\frac{TP}{TP+FN}\times 100\%$$

(4) $$F1=\frac{2PR}{P+R}$$

(5) $$\text{AP=}\int_{0}^{1}PRdR$$

Where *TP* is true positive; *FP* is false positive; *TN* is true negative; *FN* is false negative.

### 3.3 Field experiment design

**Figure 10:**
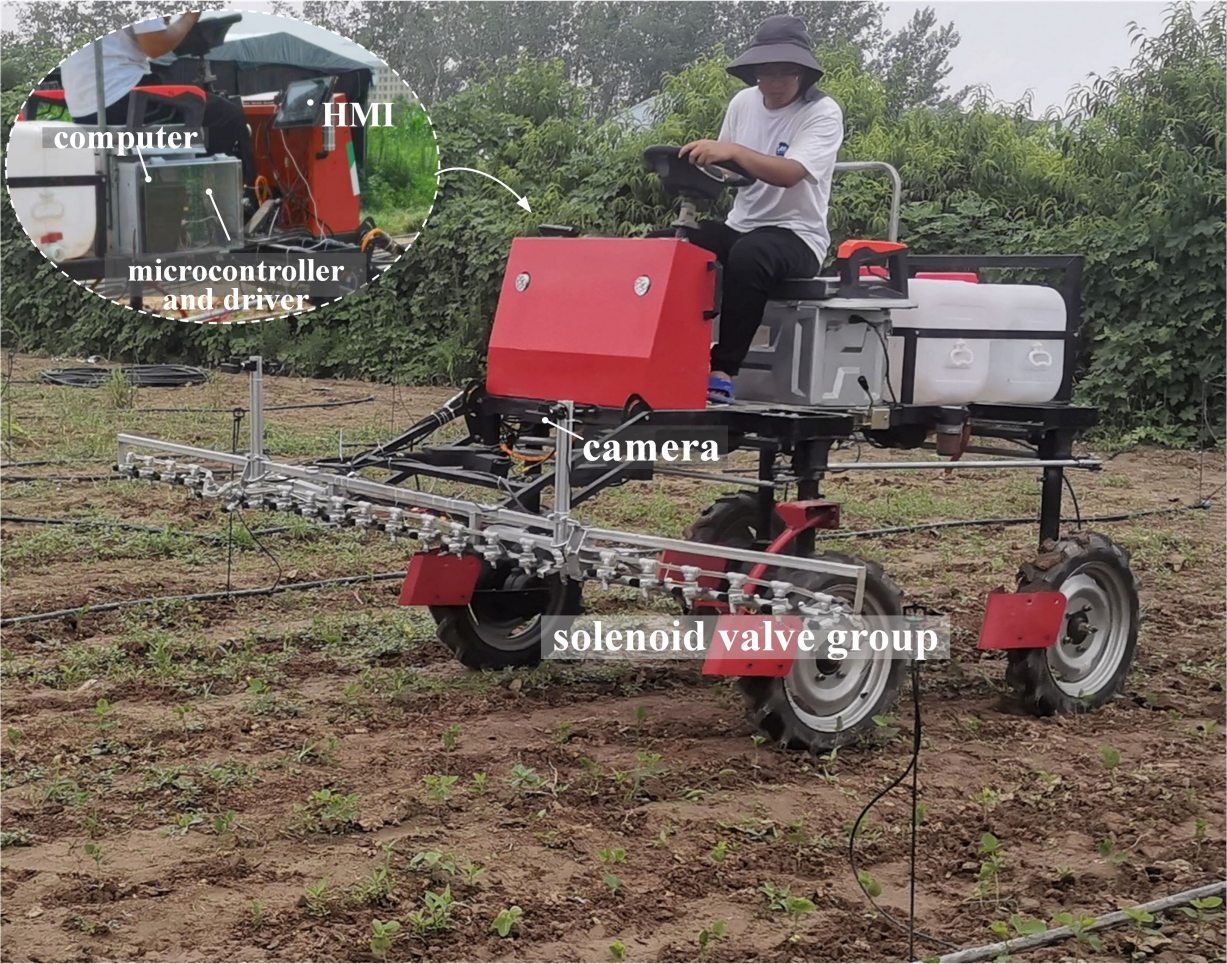
Experimental equipment.

In order to verify the accuracy of the target spraying system in the field and its effectiveness in saving chemicals, trials were conducted in Suixian County, Henan Province *(34.136°N, 115.343°E)*, where the ground plants were growing naturally. The target spraying system was mounted on an electric self-propelled upland gap spray bar sprayer, shown in **Figure 10**, with a speed meter on the sprayer to observe forward speed, and the sprayer’s battery pack was depressurized to supply power to the target spraying system. Three rectangular areas of 20×3 *m* in size were divided in the trial field as the test sampling area, and the number of weeds and spurge in the sample area and the size of the outer rectangle were counted. The speed of the sprayer was controlled at 2km/h, 3km/h, and 4km/h for the target spraying tests. A 1×1*cm* water-sensitive label, which turns red when exposed to water, is attached to each weed leaf in the test area to record whether the droplets hit the target.

## 4 Experimental results and analysis

### 4.1 Model recognition performance experiment

For the trained improved model, this study uses model evaluation metrics for computational evaluation on the test set. The experimental results show that the improved YOLOv5s model has an AP of 0.87 on the test set, an F1 score of 0.81, a model size of 7.5 MB, and an average detection time of 26.73 frames per second, which meets the requirements of real-time detection. In summary, the YOLO improved detection model proposed in this study has the advantages of high accuracy, small model size, and fast inference speed.

**Figure 11:**
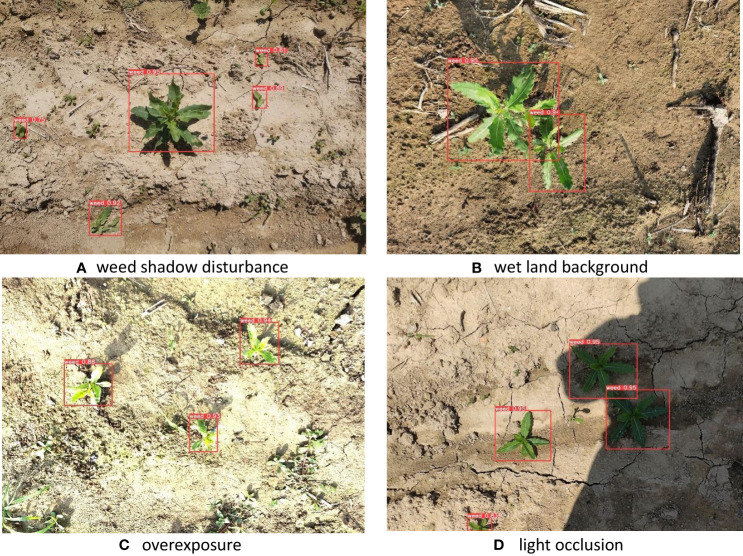
Prediction results for complex scenarios: The scenes from **A** to **D** are weed shadow disturbance, wet land background, overexposure, and light occlusion.

In practical field application scenarios, image acquisition involves many complex situations, such as overexposure, light occlusion, target shadow interference, dense targets, etc. These complex scenarios greatly affect the adaptability of the target detection algorithm. To this end, this study conducts experimental tests on image data of different scenes. **Figure 11** shows the model’s predictions for a complex scene, whereas figure a shows the weeds’ own shadow interference. Figure b shows an image with a darker-colored wet land background condition. And figure c shows an overexposed scene where the target is too bright and somewhat distorted. Figure d shows an image acquired with light obscured by a nearby tall object, making the target too bright and significantly different from the unobscured area. It can be seen from the detection results that for these complex and special scenes, the YOLOv5-MobileNet-SE target detection algorithm proposed in this study has certain adaptability and good robustness to complex scenes.

In order to better evaluate the performance of the algorithm, this study chooses to compare it with other classic deep learning object detection algorithms, YOLOv5x, YOLOv3, and Faster RCNN. For several different models, training was performed using the same training environment, while test analysis was performed on the same test platform with the same dataset. The mAP values, model sizes and recognition frame rates of the five models were obtained with a confidence level of 0.5 and an NMS threshold of 0.5. A comparison of the detection results of the different models is shown in **Table 2**.

**Table 2:** Comparison of detection results of different models.

Model	mAP0.5/%	Model size/MB	FPS
YOLOv5-MobileNet-SE	87.0	7.5	26.73
YOLOv5s	87.5	14.0	22.62
YOLOv5x	87.9	169.0	20.44
YOLOv3	87.7	120.6	23.80
Faster R-CNN	80.89	110.7	2.73

By comparing the specific parameter indicators, it can be seen that the mAP of YOLOv5-MobileNet-SE is 87.0%, the model size is 7.5M, and the FPS is 26.73. Compared with YOLOv5s, YOLOv5x, YOLOv3, Faster R-CNN, and other models, the AP index is the same, which is 0.57% lower than YOLOv5s, and 1.02% lower than YOLOv5x. For the model size, the model in this paper is the smallest among the five models, only 7.5M, which is 53.57% of the original YOYOv5s model. The FPS is the highest among the five models, at 26.73, which is 18.16% higher than YOLOv5s. It can be seen from the comparison results that the YOLOv5-MobileNet-SE model proposed in this paper has greatly optimized the size of the model and the recognition frame rate at the expense of weak accuracy, and has the best comprehensive performance, which can meet the real-time demand for lightweight models of target spray.

### 4.2 Results of field-to-target spraying experiments

**Figure 12:**
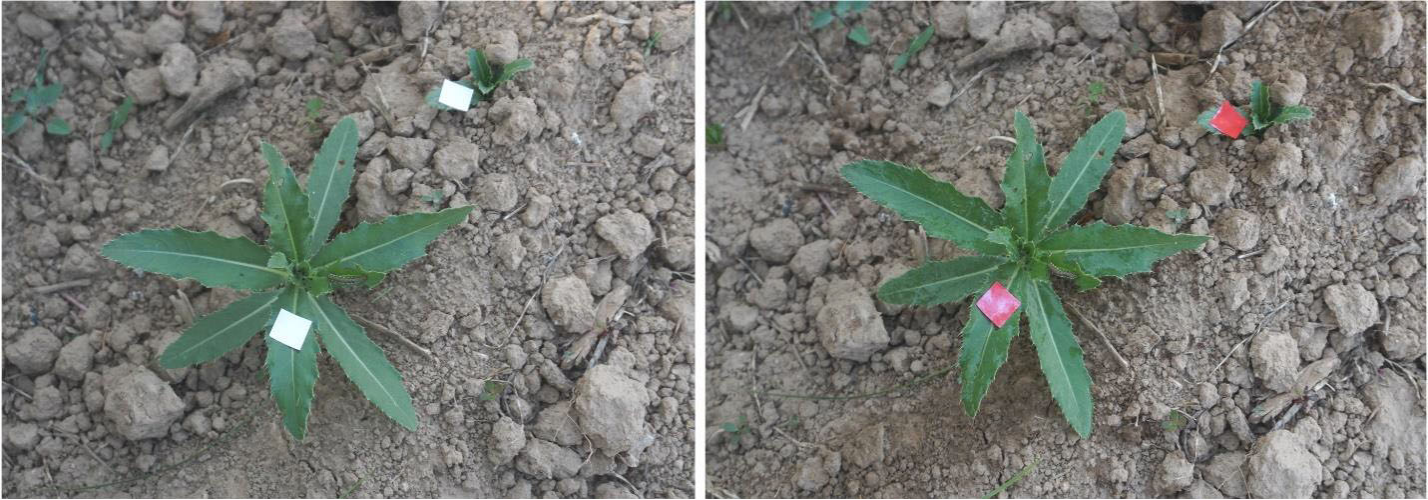
Comparison of water-sensitive label changes for target spraying experiments:the left side shows the state of the label before spraying, the right side shows the state after spraying.


In order to evaluate the comprehensive performance of the real-time target spraying system designed in this study, a field trial of target spraying was conducted. By manually observing the color change of the water-sensing label, as shown in **Figure 12**, the actual effective recognition rate, relative recognition hit rate, absolute hit rate, and other indicators of the target spray were calculated. The effective recognition rate represents the ratio of the number of weeds identified by the model to the total number of weeds in the experimental area, and the relative hit rate represents the ratio of the number of labels turning red to the number of weeds identified. The relationship between the absolute hit rate is shown in the following formula, the absolute hit rate is determined by the product of the effective recognition rate and the relative hit rate.

(6) $$w=u\times w^{\prime }$$

Where *w* is the absolute hit rate, *w’* is the relative hit rate and *u* is the effective recognition rate.

The statistical results of the target spray test at three speeds of 2km/h, 3km/h, and 4km/h are shown in **Table 3**. At a speed of 2km/h, the absolute hit rate is the highest at 90.80%. As the speed of the sprayer increased, the absolute hit rate decreased to 85.51% and 79.61% at 3km/h and 4km/h, respectively.

**Table 3:** Statistical results of the spray-on-target test.

Forward speed/km·h^-1^	Number of Cirsium setosum	Number of effective identification strains	Effective recognition rate/%	Number of hits	Relative recognition hit rate %	Absolute hit rate/%
2	87	83	95.40	79	95.18	90.80
3	145	134	92.41	125	93.28	86.20
4	103	89	86.40	82	92.13	79.61

It can be seen from the test results that the effective recognition rate and relative recognition rate of the target spray system both decrease with the increase of speed. When the forward speed increases from 2km/h to 4km/h, the effective recognition rate decreases greatly, with a decrease of 95.40%. In contrast, the relative hit rate dropped less significantly, from 95.18% to 92.13%, a decrease of 3.05 percentage points. The main reason is the uneven land in the field, the increase of speed causes the vibration of the sprayer to intensify, the quality of image acquisition reduces the effective recognition rate and also causes the nozzle mounted on the spray bar to vibrate more, resulting in a lower relative hit rate due to the misalignment of the nozzle and the target.

## 5 Conclusion

In this paper, a real-time target spraying system based on the improved YOLOv5s model is designed to achieve real-time accurate pesticide spraying in the field. Firstly, the overall design scheme of the system is proposed, including the image acquisition and recognition module, the electronically controlled spray module, and the voltage-stabilized pesticide supply module. Aiming at the problem of pressure fluctuation caused by the intermittent opening and closing of spraying nozzles on the target, a pressure-stabilizing pesticide-supplying pump with a combination of a surge tank and a pressure switch was designed. In order to realize the real-time identification of weeds in the field, the YOLOv5s model was lightweight and improved, the feature extraction backbone network was replaced with the MobileNet v3 lightweight network, and the SE attention mechanism is added to improve the accuracy of the model for small target weed identification. Then, a grille decision-making algorithm was proposed to convert the recognition results into solenoid valve opening and closing information, and at the same time to avoid the problems of short atomization time and difficult hits caused by too small identification targets. The improved YOLOv5s model is tested, and the experimental results show the accuracy and robustness of the model in complex environments. Compared with other models, the model size is only 53.57% of the YOYOv5s model, and the FPS is increased by 18.16%, realizing lightweight real-time detection. Finally, the field spray test on the target shows that the system has a high hit rate, and the spray accuracy rate is 90.80% at a speed of 2km/h, which can effectively reduce the use of pesticides and improve the effective utilization of pesticides.

## Data availability statement

The raw data supporting the conclusions of this article will be made available by the authors, without undue reservation.

## Ethics statement

Written informed consent was obtained from the individual(s) for the publication of any potentially identifiable images or data included in this article.

## Author contributions

HL, CG, and ZY contributed to the conception and design of the study. JC, JL, YS, KZ, DL, and YX organized the experimental dataset and data analysis. ZY and CG were involved in improving the recognition algorithm. HL and CG wrote the first draft of the manuscript. ZY revised the manuscript. All authors contributed to the article and approved the submitted version.
